# How I do it: judging appropriateness for TTE and TEE

**DOI:** 10.1186/1476-7120-12-22

**Published:** 2014-06-24

**Authors:** Ricardo Fonseca, Thomas H Marwick

**Affiliations:** 1 Menzies Research Institute Tasmania, 17 Liverpool St, Hobart, Tasmania 7000, Australia

**Keywords:** Appropriate use, Transthoracic echocardiography, Transoesophageal echocardiography

## Abstract

The increasing cost of healthcare is a widespread international problem to which the cost of imaging has been an important contributor. Some imaging tests are ordered inappropriately and contribute to wasted use of resources. Appropriate use criteria have been developed in the USA in order to guide test selection, but there are a number of problems, including the evidence base for these criteria and the steps that can be taken to change physician practice. A restrictive approach to test ordering is difficult to fit to the nuances of clinical presentation and may compromise patient care. We propose an alternative approach to physician guidance based on the most common markers of inappropriate testing.

## 

No management decisions in medical practice are exempt from a concept that is difficult to measure: appropriateness. In common parlance, an appropriate choice is one that which is suitable or proper in the circumstances, but this is surprisingly different from the medical definitions. The concept of appropriateness defined by the RAND/UCLA methodology in the 1980’s was the cornerstone for developing the first attempt at appropriate use criteria (AUC). That concept suggested that “an appropriate procedure in one in which the expected health benefit (e.g, increased life expectancy) exceeds the expected negative consequences (e.g., mortality, morbidity, anxiety, pain, time lost from work) by a sufficiently wide margin that the procedure is worth doing, exclusive of cost” [1,2].

The adaption of this concept to cardiac imaging led to an appropriate test being defined as “one in which the expected incremental information, combined with clinical judgement, exceeds the expected negative consequences (risks of the procedure i.e. radiation or contrast exposure and the downstream impact of poor test performance, such as delay in diagnostic (false negatives) or inappropriate diagnosis (false positives)) by a sufficiently wide margin for specific indication that the procedure is generally considered acceptable care and a reasonable approach for the indication” [3]. Because of the low risk of imaging, there are many circumstances in where this definition seems to be insufficient – the risk is almost zero so the balance of benefit and risk is positive, but the information obtained is still inadequate to justify performance of the test. A new definition overcomes these concerns by framing the decision in the context of a consensus about “reasonable care” [4], and resource utilization “The concept of appropriateness, as applied to health care, balances risk and benefit of a treatment, test, or procedure in the context of available resources for an individual patient with specific characteristics” [5]. Importantly, it is now acknowledged that AUC should provide guidance to supplement the clinician’s judgment, rather than being prescriptive.

## Motivations to the definition of appropriate use criteria

**Figure 1:**
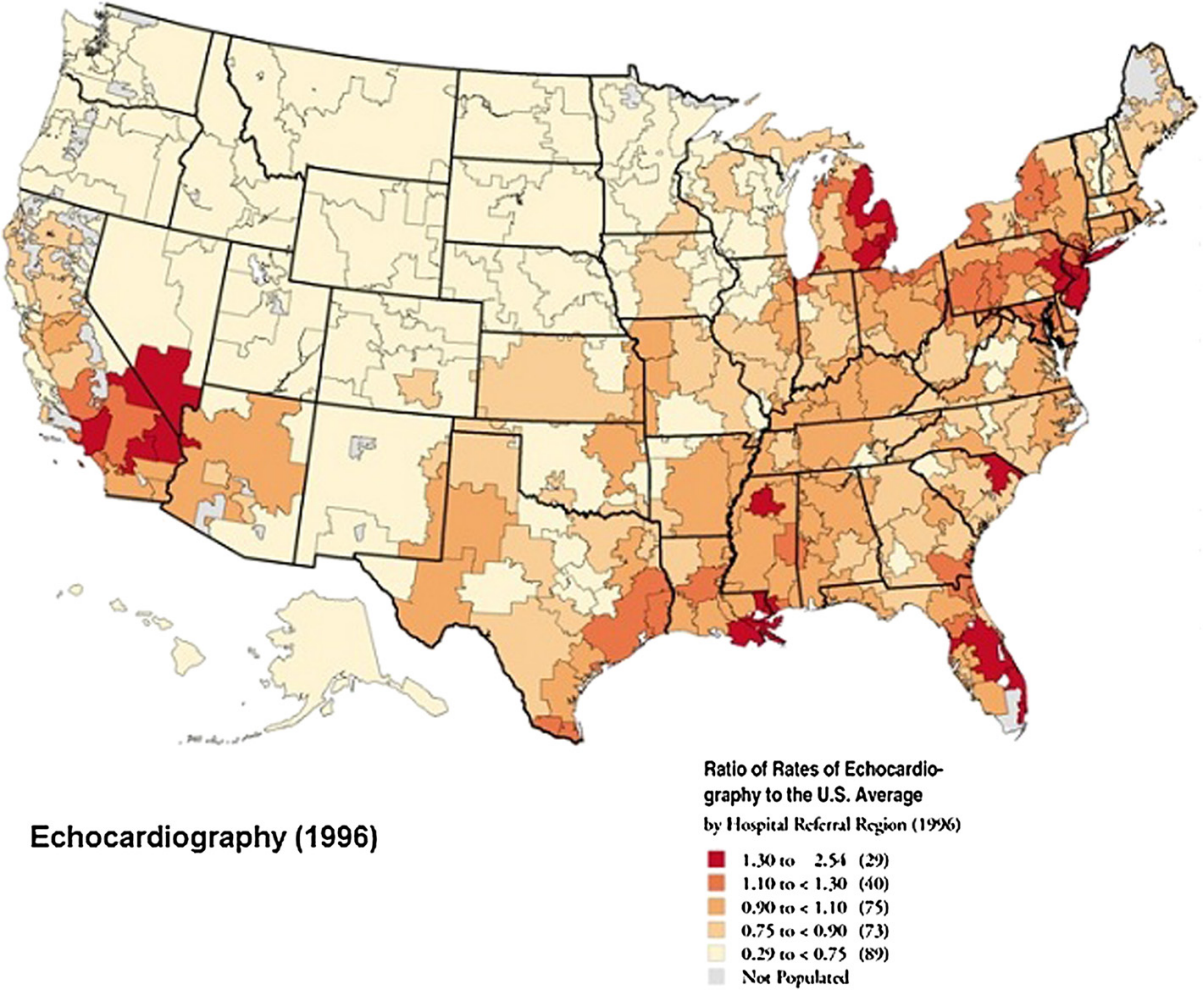
**Differences in the use of echocardiography in the US in 1996.** Regional variations by hospital referral region, expressed as a ratio to the US average. From Wennberg D, et al. The Dartmouth Atlas of Cardiovascular Health Care. P65. 1999 [11].

While the risk of harm with inappropriate intervention was an important motivator to the application of AUC, the focus on appropriate use in imaging is mainly rooted in resource utilization and medical expenditure. The contribution of imaging to the medical budget started to be highlighted in the United States >20 years ago. At this time, the Medicare Payment Advisory Commission (MedPAC) showed a 10%/year increase of spending for cardiac imaging between 1999 and 2002, when the average growth per year of all services was 5.2% [6]. This continued throughout the following decade – imaging payments to Cardiologists in 2000 were US$1.6 billion, increasing to US$5.1 billion in 2006 [7]. Contributors to this growth included the rapid proliferation of imaging machines, limited experience with new imaging modalities among non-specialists, automated referral pathways, poor quality of imaging (requiring repetition) and defensive medicine [8]. Differences in the use of imaging among regions supported the contention that the selection of imaging test was discretionary rather than disease-related [9-11] (Figure 1).

## Development and application of appropriate use criteria

One of the responses to the overuse of imaging was the development of AUC. The American College of Cardiology Foundation (ACCF) along with other medical associations formed the Appropriateness Criteria Working Group (now called ACCF AUC Task Force) [4], which used a modified RAND/UCLA methodology [1,2] to elaborate the criteria. After the review of possible indications, an expert rating panel determined if an indication was appropriate, uncertain or inappropriate (now called appropriate, may be appropriate and rarely appropriate by the new methodology) [3,4].

The first AUC (for SPECT) were launched at the end of 2005 and the first transthoracic (TTE) and transesophageal (TEE) echocardiography AUC document was released two years later [12,13]. Stress echocardiography (SE) was not included in the first version of the echocardiography AUC [14], but these criteria were merged in the 2011 version [15]. The AUC continue to evolve, and criteria for multimodality cardiac imaging and the re-definition of “inappropriateness” represent recent changes [4,16].

While the AUC have become a cornerstone of the efforts to improve quality in the USA, their uptake in other jurisdictions has been less enthusiastic. The current criteria have a number of disadvantages [17-43];

1) The AUC have been defined by consensus. The scientific basis of some AUC is weak, with level of evidence B or C.

2) AUC represent a compilation of indications but not all situations in which an echocardiogram could be performed are addressed. Although some studies of AUC indicate all tests to have been classified [18,19,25,31,32,37,38], in reality, several indications are often present in the same patient. Retrospective audit may be especially problematic, as the reason for requesting an echocardiogram is often inadequately detailed in the medical records.

3) Conversely, several recommendations for echocardiography in current practice guidelines (not just in echocardiography but for disease entities) lack counterparts in the AUC. For example, a class I recommendation is given for follow-up or surveillance after surgery of masses known to have a high likelihood of recurrence (eg myxoma [44]). The AUC classification of “suspected cardiac mass” – or even screening – does not cover the described scenario.

4) The application of AUC to patient selection may be problematic as an audit tool. When an appropriate indication is required to order the test at point-of-service, the referring clinician may list a co-existing appropriate indication rather than the actual clinical problem (which may be of inappropriate). This is particularly likely when the proportion of inappropriate tests is assessed as part of the echocardiography accreditation process.

After 7 years of using the AUC for echocardiography (TTE and TEE), there are concerns about the real impact of the AUC on physician ordering behaviour [45]. The literature seems to show a similar proportion of inappropriate testing, in spite of experience, educational campaigns and close follow-up. Moreover, the correlation between appropriateness and clinical impact has not been well studied [31].

## Application of AUC in daily practice

We do not favour the use of AUC as a “gatekeeper” to echocardiography. Rather, we see the AUC provide a yardstick to permit three means of improving appropriateness - education, guidance at point-of-care and laboratory-based audit;

i). Education: Although educational interventions seem to be a logical approach, the results of heterogeneous attempts have been contradictory. On the one hand, for instance, an educational campaign consisted in lectures, a pocket card with the AUC and feedback showed encouraging results as one of the successful tools for improving appropriateness [23]. On the other hand, similar projects focused in physician education and feedback [46,47], did not show improvement. The AUC are an excellent starting point in this respect. Essential parts of educational campaigns include lectures, pocket cards and feedback.

ii). Control in point-of-care: The use of prior authorisation protocols through a Radiology Benefit Manager (RBM) is widely used to control access to expensive tests of limited availability, such as positron emission tomography and cardiac magnetic resonance, although its efficiency and effectiveness have been questioned [47]. The use of AUC at point of care involves ordering physicians in the attempt to decrease inappropriate tests. In order to facilitate this, friendly electronic tools have been invented to help clinicians to choose “appropriately” at the point-of-order [24]. Recent work has proposed that this practice is of equivalent efficacy to the use of the RBM [48], with greater efficiency and better preservation of the autonomy of the attending physician. Incorporation with an electronic ordering process can inform the clinician about appropriateness when the test is requested. The risk of both AUC and RBM are that other appropriate (but inactive) clinical problems that can be used to have a test approved to address an inappropriate question.

iii). Laboratory-based audit: We have focused on this because of the limitations of the above two methods. Laboratories are potentially more motivated than requestors because of the reputational and economic risk of high levels of inappropriate use. While we acknowledge that the audit process can be problematic in private practice, as the locus of control is with the referring doctor, it is important to consider that the laboratory will be held responsible for the performance of inappropriate tests and the consequence of more inappropriate tests will be less reimbursement. In this setting, it seems likely that some investment into auditing this process will be reasonable. Inevitably, urgent echocardiograms and communication problems represent scenarios where the process is challenging, but if appropriateness is to be audited, we would suggest that defining the “at risk” study for inappropriateness (see below) is a means of improving the efficiency of this process from needing to audit 100% of requests to audit of the ~15% of requests that are included in this list. The additional scrutiny given to these requests does not necessitate individual contact with the referring physician in all cases.

## Screening imaging requests for appropriateness

**Figure 2:**
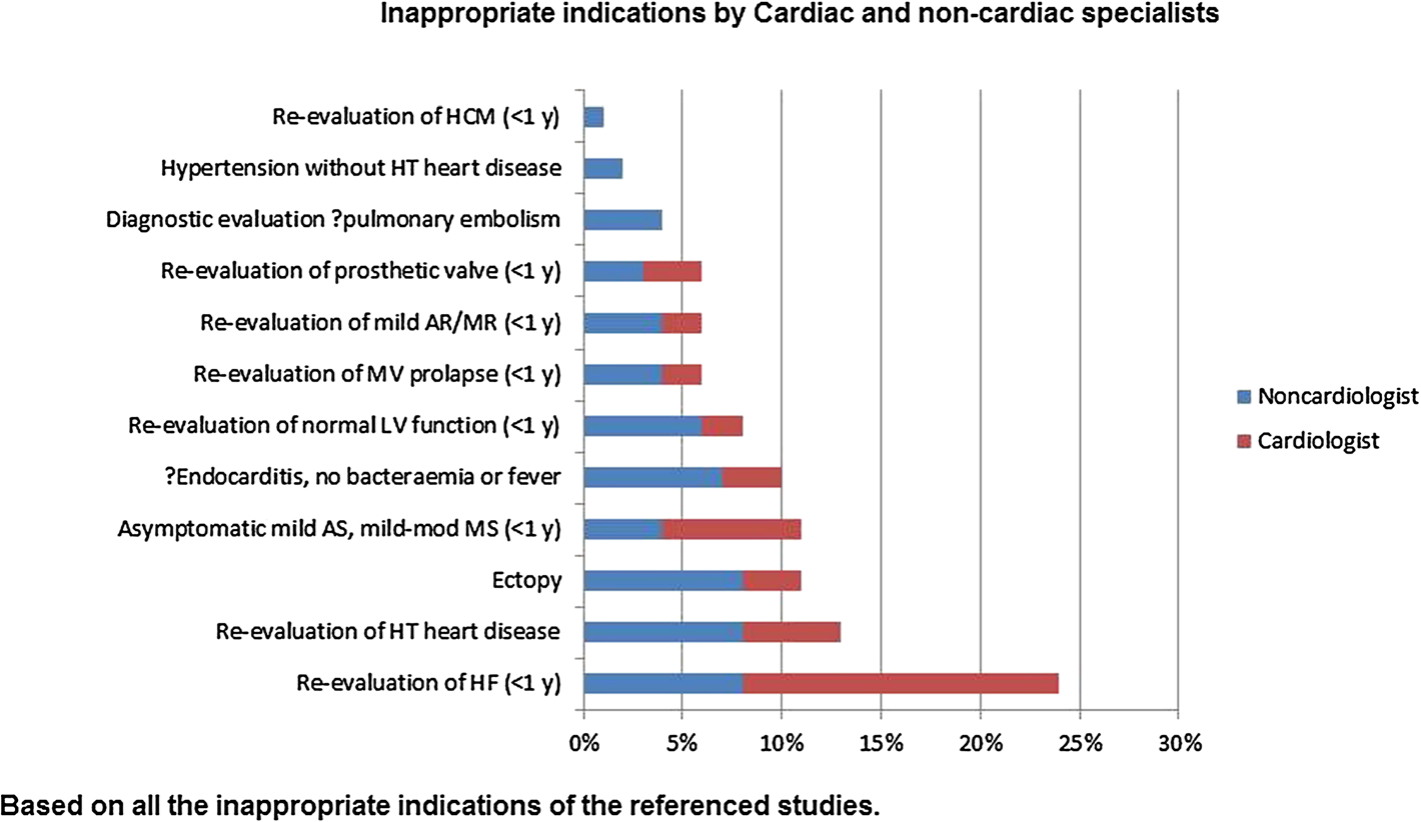
**Major causes of inappropriate echocardiography.** Proportions of inappropriate tests (x axis) ordered by cardiologists (red) and non-cardiologists (blue). Modified from Ward RP et al. [39].

If the strategy of laboratory-based audit is selected, a simple screening process is required for the thousands of requests which are submitted to the laboratory every year. Our approach has been to base this around the indications which generate the greatest numbers of inappropriate tests in the 2011 AUC for echocardiography (TTE and TEE, but not including stress, Figure 2) [15]. These are related to routine surveillance, evaluation of symptoms without other symptoms/signs of cardiac disease and low pretest probability of endocarditis [18,20,21,23,33,34,36,41]. Other situations include a suspicion of pulmonary embolism, when the exam would not change management, and when a test is ordered by non-cardiologists.

**Figure 3:**
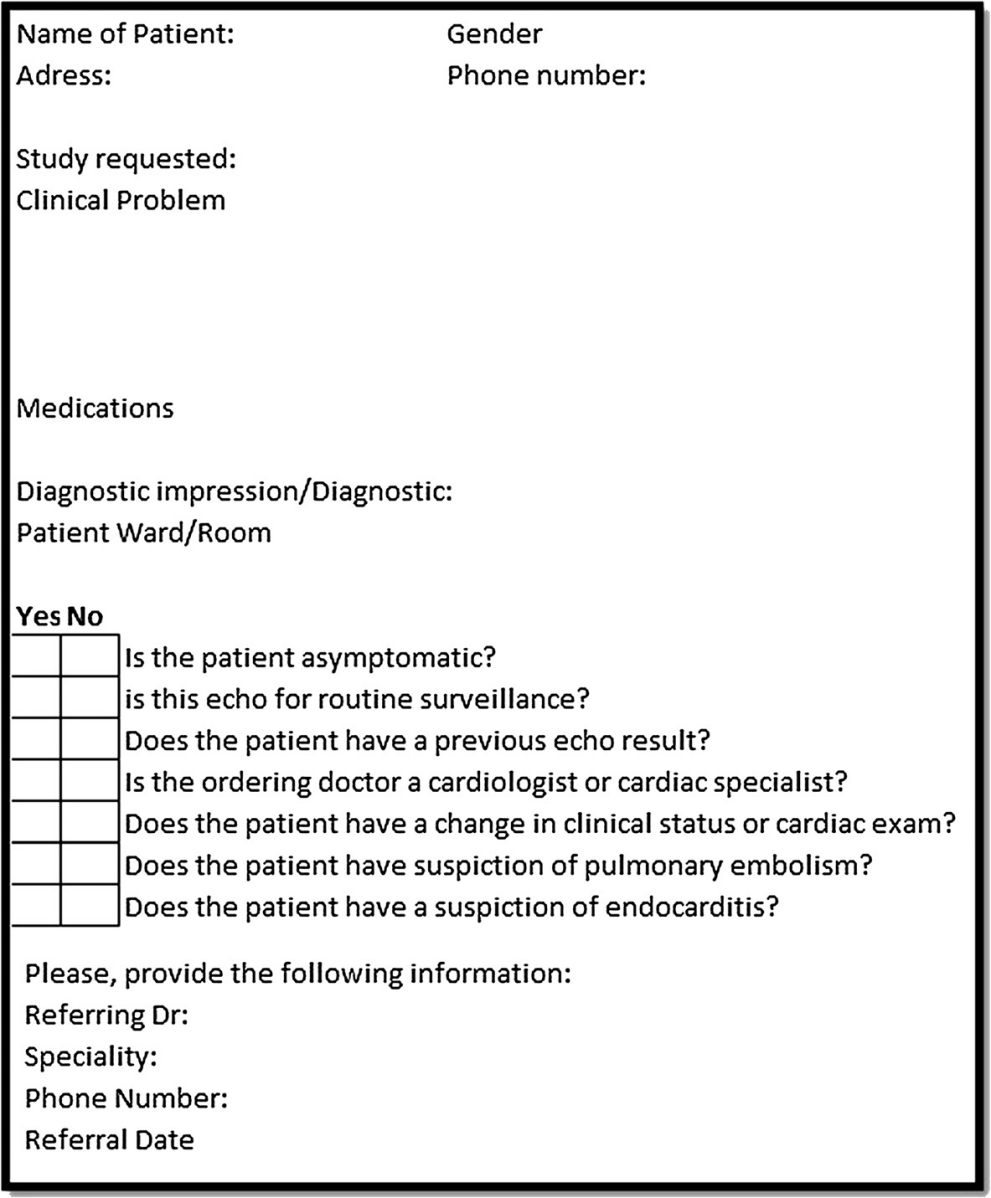
**Proposed checklist to discriminate possible inappropriate orders.** A simplified check-list to be reviewed at point of service, as a prompt to seeking clarification from the referring physician.

Routine surveillance is the most common inappropriate indication for TTE. The most common situations of inappropriate repeat imaging of ventricular function include assessment in patients with known CAD and no change in clinical status or cardiac exam [34,41], systemic hypertension without symptoms or signs of hypertensive heart disease [20], and within a year of previous testing in heart failure (systolic or diastolic) when there is no change in clinical status or cardiac exam [20,34]). A very common situation in patients with nonspecific symptoms includes patients with lightheadedness/presyncope without other symptoms) [23,41]. Common valve-related indications include <3 year after prosthetic valve implantation in the absence of known or suspected valve dysfunction [33], and evaluation of infective endocarditis when there is transient fever without evidence of bacteremia [23] or new murmur or transient bacteraemia with a pathogen not typically associated with endocarditis. For transoesophageal echocardiography, the most common inappropriate indications are related to endocarditis with low pretest probability and routine use of TEE when a diagnostic TTE is reasonably anticipated to resolve all concerns [21].The availability of this information on the characteristics of inappropriate tests has enabled the development of a checklist to identify studies where a discussion regarding the merits of testing can be initiated from the laboratory (Figure 3).

## Conclusions

Judging appropriateness in echocardiography is a process based on knowledge, experience, information, resources and the real desire to provide an adequate service to the patient. It does not sit well with formulaic approaches based on uncritical application of AUC. Importantly, it is now acknowledged that AUC should provide guidance to supplement the clinician’s judgment, rather than being prescriptive [5].

Although the audit process described above helps to strengthen the application of the AUC, it is difficult to control the problems associated with self-referral and the veracity on the part of ordering physicians. In our opinion, the optimal approach requires dialogue between treating physicians, cardiologists and sonographers. The perfect tool has not yet been designed, but a process that flags discussion at the point of imaging may be more effective than a gatekeeper at the point of ordering the test.

## Abbreviations

ACCF: American college of cardiology foundation; AUC: Appropriate use criteria; CAD: Coronary artery disease; MedPAC: Medicare payment advisory commission; RAND/UCLA: RAND corporation/university of California Los Angeles; RBM: Radiology benefit manager; SE: Stress echocardiography; SPECT: Single photon emission computed tomography; TTE: Transthoracic echocardiography; TEE: Transesophageal echocardiography.

## Competing interests

The authors declare that they have no competing interests.

## Authors’ contributions

RF; contributed to conception/design, gathered and interpreted data and wrote the paper, approved final version and agrees to be accountable for content. THM; contributed to conception/design, gathered and interpreted data, edited the paper, approved final version and agrees to be accountable for content.
